# Structural and Functional Insights into Viral Programmed Ribosomal Frameshifting

**DOI:** 10.1146/annurev-virology-111821-120646

**Published:** 2023-06-20

**Authors:** Chris H. Hill, Ian Brierley

**Affiliations:** 1 York Structural Biology Laboratory, York Biomedical Research Institute, Department of Biology, University of York, York, United Kingdom; 2 Department of Pathology, University of Cambridge, Cambridge, United Kingdom

**Keywords:** ribosome, translation, frameshift, pseudoknot, SARS-CoV-2, HIV

## Abstract

Protein synthesis by the ribosome is the final stage of biological information transfer and represents an irreversible commitment to gene expression. Accurate translation of messenger RNA is therefore essential to all life, and spontaneous errors by the translational machinery are highly infrequent (~1/100,000 codons). Programmed −1 ribosomal frameshifting (−1PRF) is a mechanism in which the elongating ribosome is induced at high frequency to slip backward by one nucleotide at a defined position and to continue translation in the new reading frame. This is exploited as a translational regulation strategy by hundreds of RNA viruses, which rely on −1PRF during genome translation to control the stoichiometry of viral proteins. While early investigations of −1PRF focused on virological and biochemical aspects, the application of X-ray crystallography and cryo–electron microscopy (cryo-EM), and the advent of deep sequencing and single-molecule approaches have revealed unexpected structural diversity and mechanistic complexity. Molecular players from several model systems have now been characterized in detail, both in isolation and, more recently, in the context of the elongating ribosome. Here we provide a summary of recent advances and discuss to what extent a general model for −1PRF remains a useful way of thinking.

## 1 Introduction

Programmed −1 ribosomal frameshifting (−1PRF) is a translational control mechanism that facilitates the coordinate expression of two (or more) proteins from a single messenger RNA (mRNA), often at a defined ratio (reviewed in 1–5). During elongation, ribosomes decode the mRNA in triplet steps and maintain the reading frame accurately. However, in −1PRF, the ribosome is induced at high efficiency to shift one nucleotide backward into an overlapping reading frame and translate an entirely new sequence of amino acids, essentially fusing the zero- and –1-frame polypeptides (6). It is predominantly an RNA virus phenomenon, often in viruses of clinical, veterinary, and agricultural importance (1, 7), but has also been documented in certain DNA bacteriophage, in replicating elements such as insertion sequences and transposons, and in a small number of cellular genes of both prokaryotes and eukaryotes (1). Maintaining a precise efficiency of frameshifting has been shown to be critical to the replication of several viruses, including human immunodeficiency virus 1 (HIV-1) (8, 9), Rous sarcoma virus [RSV (10)], the yeast double-stranded RNA virus [L-A (11)], severe acute respiratory syndrome coronavirus (SARS-CoV) (12, 13), Venezuelan equine encephalitis virus (14), and bacteriophage lambda (15). In retroviruses, frameshifting at the overlap of the *gag* and *pol* open reading frames (ORFs) permits expression of the viral Gag-Pol polyprotein and sets a defined cytoplasmic Gag:Gag-Pol ratio (typically 20:1) optimized for virion assembly and packaging of reverse transcriptase. In RNA viruses, frameshifting allows expression of viral replicases; in coronaviruses, for example, ~50% of ribosomes translating *ORF1a* frameshift into the replicase-encoding *ORF1b* (16–18), with a resultant pp1a:pp1ab ratio of 1:1. Frameshifting is thus a logical target for antiviral intervention, and several in vitro studies support this hypothesis (see Section 4).

Recently, high-resolution structures of the molecular players of −1PRF have been solved, permitting the development of more refined models of the process and providing insights for targeted therapies. These studies have benefited from transformative technological advances in structural biology, ribosome profiling, and the vast databases developed by the highly active ribosome structure and translation communities. The increased scrutiny of coronavirus replication mechanisms during the severe acute respiratory syndrome coronavirus 2 (SARS-CoV-2) pandemic has also been seminal in driving recent studies on −1PRF. Here we review the structural biology of frameshifting, discuss models of the process, and consider the next steps in the field.

## 2 Molecular Players

**Figure 1:**
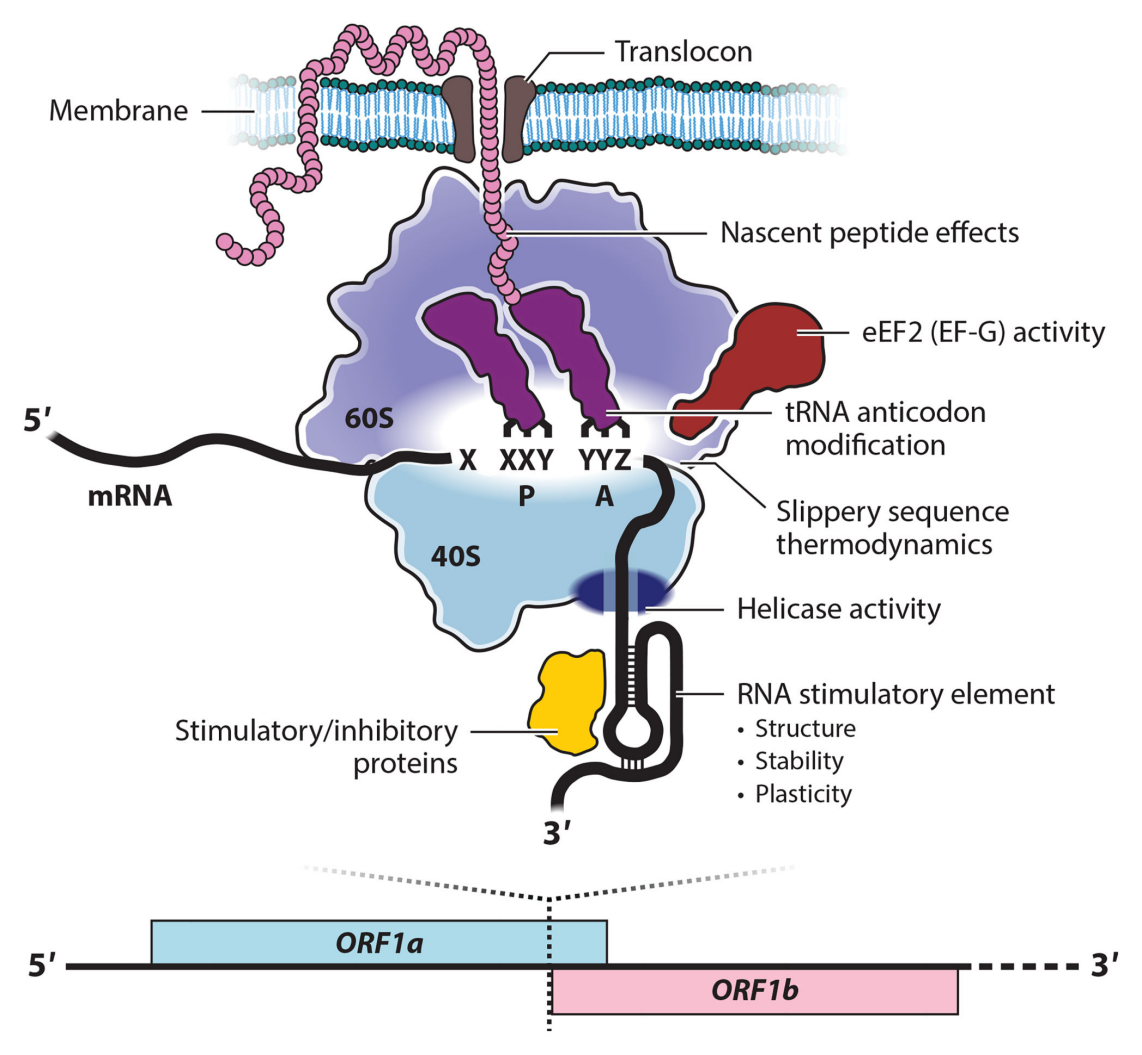
Schematic diagram summarizing the molecules and principles governing viral −1PRF. The eukaryotic ribosome is depicted in the act of translating a frameshift-stimulatory signal in an mRNA. Key players with frameshift-modulatory activity are labelled. Abbreviations: −1PRF, programmed −1 ribosomal frameshifting; A, aminoacyl-site; mRNA, messenger RNA; ORF, open reading frame; P, peptidyl-site; tRNA, transfer RNA.

The mRNA signal for frameshifting is typically bipartite, comprising a slippery sequence, where the ribosome changes frame, separated by a spacer region [usually 5–9 nucleotides (7)] from a downstream stimulatory mRNA structure (19). These and other molecular players in −1PRF are shown in Figure 1, and key features are detailed below.

### 2.1 Base Pairing in the Decoding Center of the Ribosome

One of the key factors that influences −1PRF is the interaction between the slippery sequence codons and the transfer RNAs (tRNAs) that decode them on the ribosome. Below, we outline the features that define an efficient slippery sequence and debate the role of tRNA anticodon modification in −1PRF.

#### 2.1.1 The slippery sequence

The slippery sequence is a heptanucleotide stretch containing two homopolymeric triplets and conforms in the majority of cases to X_XXY_YYZ (zero frame delineated by underscores; in eukaryotes, X is any nucleotide, Y is usually A or U, and Z is any base but G). Here, ribosome-bound peptidyl- (P) and aminoacyl- (A) site tRNAs slip into the −1 frame (to XXX_YYY), perhaps simultaneously, with translation continuing after the frameshift. The slippery sequence is permissive for the formation of post-slippage codon-anticodon pairs at the first two positions of each codon, and wobble base pairing does not appear to be required. The magnitude of the frameshift is influenced by several factors, one of which is the stability of codon-anticodon interactions, particularly in the ribosomal A-site. At viral signals, the A-site triplet is rarely CCZ or GGZ, consistent with the idea that stronger zero-frame base pairing decreases the likelihood of the tRNA unpairing and reassociating in the −1 frame (1). Further, in eukaryotes, G is avoided at the end of the slippery sequence, probably due to the presence of tRNA isoacceptors that can form a stable G-C pair at the wobble base preslippage, restricting frameshifting. Thermodynamic modeling supports a correlation between −1PRF efficiency and the free energy of codon:anticodon base pairing on the slippery sequence (20). Kinetic analysis indicates that the tRNAs may spontaneously and reversibly fluctuate between reading frames, establishing an equilibrium based on the relative free energies of pairing in the zero and −1 frames (21).

#### 2.1.2 Transfer RNA anticodon loop modifications

**Figure 2:**
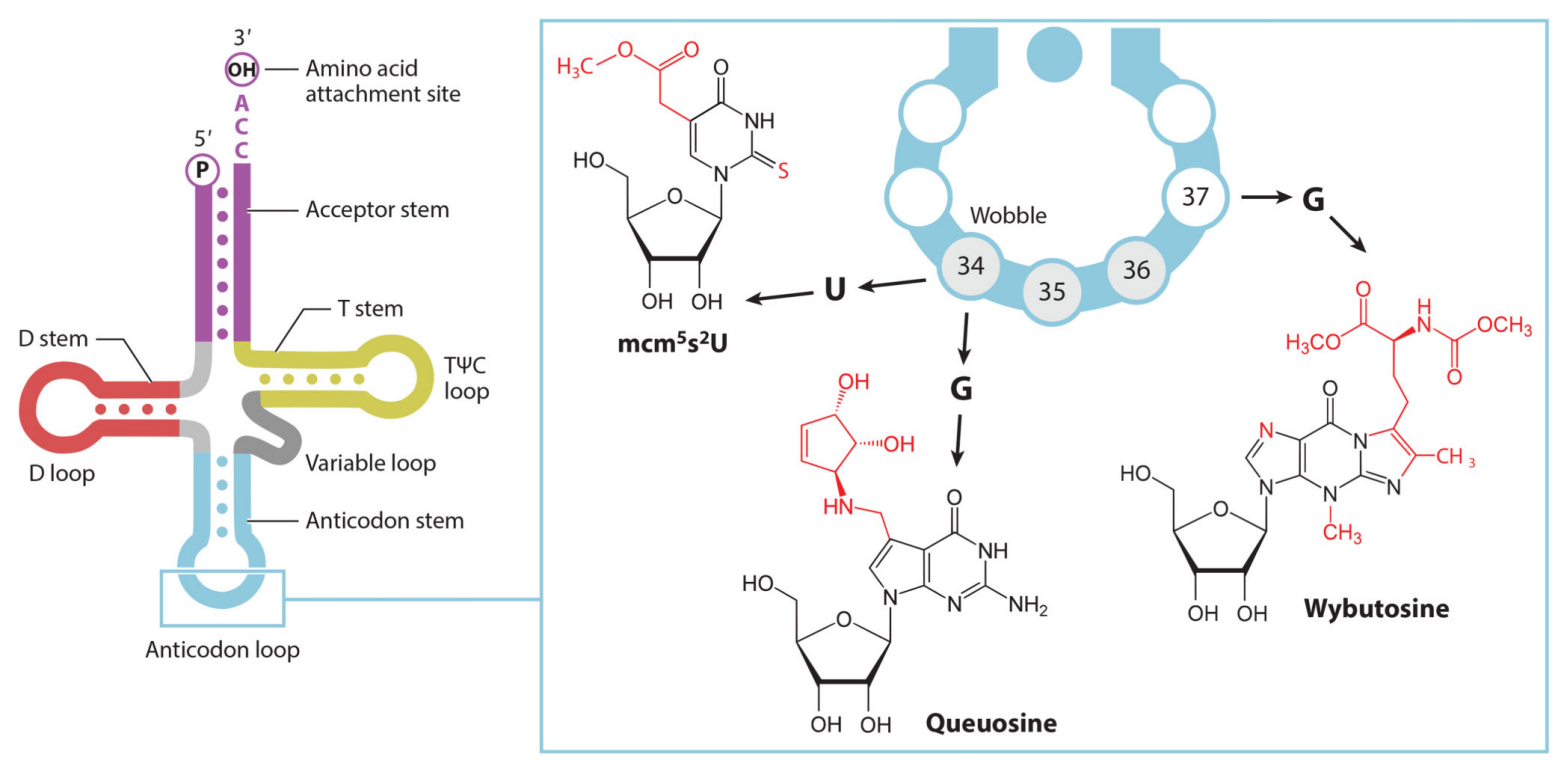
Anticodon loop modifications associated with −1PRF. A schematic of a tRNA is shown on the left, with a zoom-in of the anticodon loop on the right. Modified wobble-bases include 5-*mcm5s2U* and Q. The modified base yW is found adjacent to the anticodon (residues 34–36). Abbreviations: −1PRF, programmed −1 ribosomal frameshifting; *mcm5s2U*, 5-methoxycarbonylmethyl-2-thiouridine; Q, queuosine; tRNA, transfer RNA; yW, wybutosine.

Noticeably, prevalent slippery sequence A-site codons such as AAC, UUU, and UUA are decoded (in eukaryotes) by tRNAs with base modifications at the wobble position (position 34) or adjacent to the anticodon (position 37) (Figure 2).

An early idea in the field was that −1PRF might be stimulated by tRNAs with a hypomodified anticodon loop, perhaps induced by virus infection, as such tRNAs may be freer to move around at the decoding site (22). Supporting this, in assays of the human T cell lymphotrophic virus I (HTLV-I) *gag/pro* and L-A virus −1PRF signals, removal of the wybutosine (yW) modification immediately 3′ of the anticodon of tRNA^Phe^ (decoding UUU and UUC) increased frameshifting (23, 24). In contrast, elimination of the Q34 (queuosine) modification in mammalian tRNA^Asn^ (decoding AAC and AAU) had no effect on frameshifting at the coronavirus infectious bronchitis virus (IBV) *1a/1b* signal (25). It is now thought that anticodon-loop base modifications most likely influence frameshifting through their effect on the stability of codon-anticodon interactions (reviewed in 5). How tRNA modification might influence virus replication, via modulation of −1PRF, has received less attention. By mutation of the host 2-thiouridine biosynthesis pathway, Maynard and colleagues (26) demonstrated that bacteriophage lambda replication is negatively affected through alteration of the stoichiometry of the lambda tail proteins gpG and gpGT, expressed by −1PRF (27). They found that hypomodified tRNA^Lys^ (modified anticodon is 5′ mnm^5^s^2^UUU 3′; decodes AAA and AAG) with decreased thiolation was more prone to frameshift on the G_GGA_AAG slippery sequence, decreasing the gpG:gpGT ratio and reducing lambda phage production. Rak and colleagues (28) recently described dynamic changes in tRNA modification and abundance during T cell activation, including a dramatic reduction in yW content. The authors propose that such hypomodification may support rapid protein synthesis during T cell activation, at the expense of decoding accuracy, and might potentially be exploited by HIV-1 to increase frameshifting (on slippery sequence U_UUU_UUA) during replication. In T cell lines, the tRNA^Leu^ isoacceptor that decodes UUA is rare, leading to a stimulation of frameshifting (29). Hypomodification of tRNA^Phe^ decoding UUU may also augment the process.

### 2.2 RNA Stimulatory Elements

A second critical element in −1PRF is the presence of a stimulatory element of some kind. Below, we discuss stimulatory RNAs, their structural features, and the attendant mechanistic implications.

#### 2.2.1 Pseudoknots

**Figure 3:**
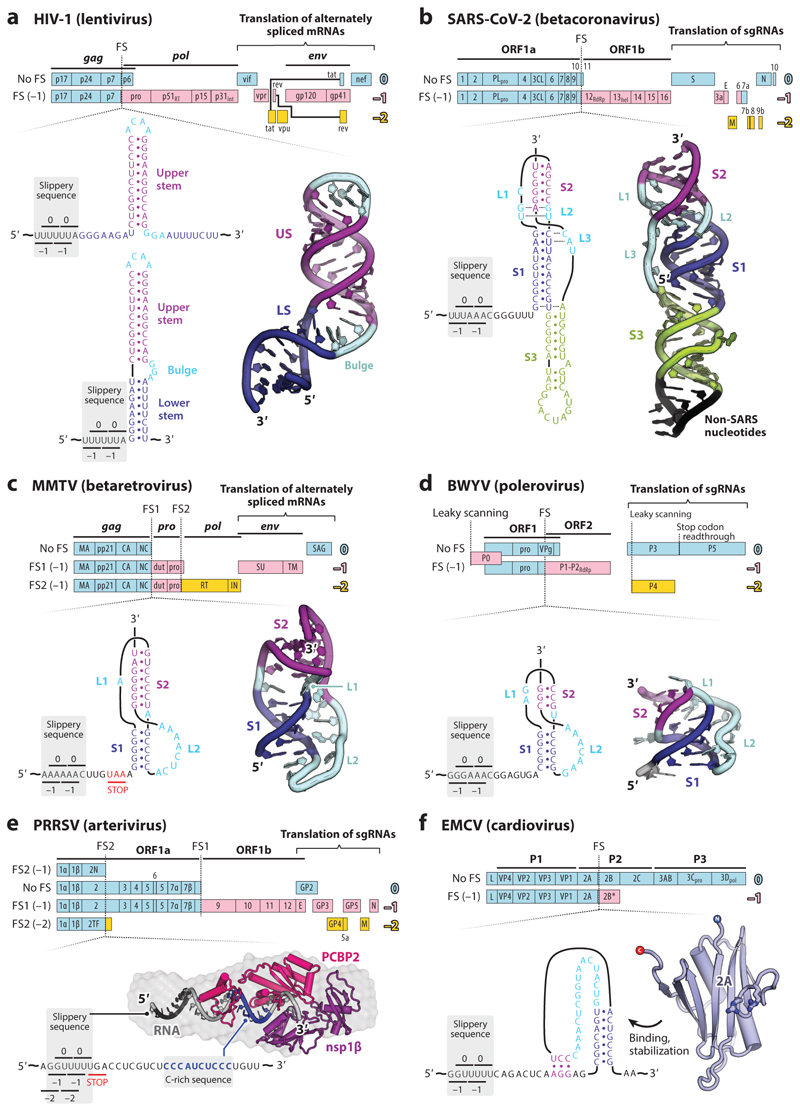
Illustrating the structural diversity of −1PRF stimulatory elements in canonical (*a–d*) and protein-stimulated (*e*,*f*) frameshifting. The upper part of each panel shows a summary of the viral translation products. The genomic frame of each protein relative to the main ORF (0; *blue*) is indicated. In addition to PRF, products encoded in other reading frames may be accessed by alternative splicing, translation of subgenomic RNAs, leaky scanning, or stop codon read through. The lower part of each panel shows a topological diagram and representative 3D structure of stimulatory elements, color coded by feature. In panel *e*, the proposed arrangement of components is informed by biochemical experiments and docking into a SAXS envelope (*gray*). In panel *f*, the structure of the 2A protein is known but the tertiary structure of the cognate RNA element is unknown. Abbreviations: −1PRF, programmed −1 ribosomal frameshifting; BWYV, beet western yellows virus; EMCV, encephalomyocarditis virus; FS, frameshift site; HIV-1, human immunodeficiency virus 1; MMTV, mouse mammary tumor virus; mRNA, messenger RNA; nsp, nonstructural protein; ORF, open reading frame; PCBP, poly(C) binding protein; PRRSV, porcine reproductive and respiratory syndrome virus; SARS, severe acute respiratory syndrome; SARS-CoV-2, severe acute respiratory syndrome coronavirus 2; SAXS, small-angle X-ray scattering; sgRNA, subgenomic RNA.

Slippery sequences alone can stimulate −1PRF to levels substantially higher than the natural error rate of frame maintenance [~1 error per 10^5^ decoding events (see 4)], but high-level frameshifting (>1%) almost always requires a stimulatory motif. This generally takes the form of an RNA structure located a few nucleotides downstream of the slippery sequence: a stem-loop (e.g., Figure 3*a*) or more commonly an RNA pseudoknot (19, 30, 31) (e.g., Figure 3*b–d*). All pseudoknots possess, by definition, two base-paired stems (S1 and S2) connected by single-stranded loops [L1, L2, and L3 (as defined in 32; see 33)], but what stands out from a plethora of analyses is the variety of tertiary structure features on display (34). This hints that −1PRF is promoted by complex mRNA geometry.

It has long been speculated that frameshifting is linked to an unwinding activity of the ribosome, with the stimulatory RNA presenting an unusual topology that resists this process. The prokaryotic 70S ribosome can itself act as a helicase to unwind mRNA secondary structures before decoding, with the active site located between the head and shoulder of the 30S subunit (35–37). Ribosomal proteins uS3, uS4, and S5 that line the mRNA entry tunnel are implicated in the helicase activity (35), with uS3 and eS30 likely to form important elements of the mammalian 80S helicase (38). A common pseudoknot feature that could account for an intrinsic resistance to unwinding is the minor groove triplex interactions between S1 and the crossing L3, first observed in the pseudoknot of the polerovirus beet western yellows virus (39) (Figure 3*d*) and subsequently in related *Solemoviridae* (40–42) and the *gag/pro* pseudoknot of simian retrovirus 1 (SRV-1) (43). The S1-L3 RNA triplex is likely to be the first feature encountered by the elongating ribosome, and the presence of the third strand might confound unwinding (37). However, a variant of the sugarcane yellow leaf virus pseudoknot containing an inactivating point mutation (C27A) retains the same overall triplex structure as the wild-type RNA, so triplex topology alone is insufficient to induce frameshifting (44).

The C27A mutation is located at the junction between constituent pseudoknot helices, highlighting the functional importance of this area. In several structures, base triples, base quadruples, and loop-loop Hoogsteen base pairing at the S1/S2 junction have been noted, as well as distortions such as over-rotation of the stems, helical displacement, and bending (44, 45) (Figure 3*b–d*). Nuclear magnetic resonance (NMR) and mutational analysis of the mouse mammary tumor virus (MMTV) *gag/pro* pseudoknot indicates a requirement for a specific bent conformation, brought about in part by the presence of an intercalated, unpaired L1 adenosine residue between the two stems (46, 47) (Figure 3*c*). However, closely related pseudoknots with coaxially stacked stems can stimulate high levels of frameshifting (43, 48), and it now seems likely that the specific interactions and resultant architectures of the helical junction required for frameshifting are strongly context dependent. Nevertheless, there is no doubt that the junction conformation is crucial to function and may present a kinetic or thermodynamic barrier to unwinding (42, 44, 49).

**Figure 4:**
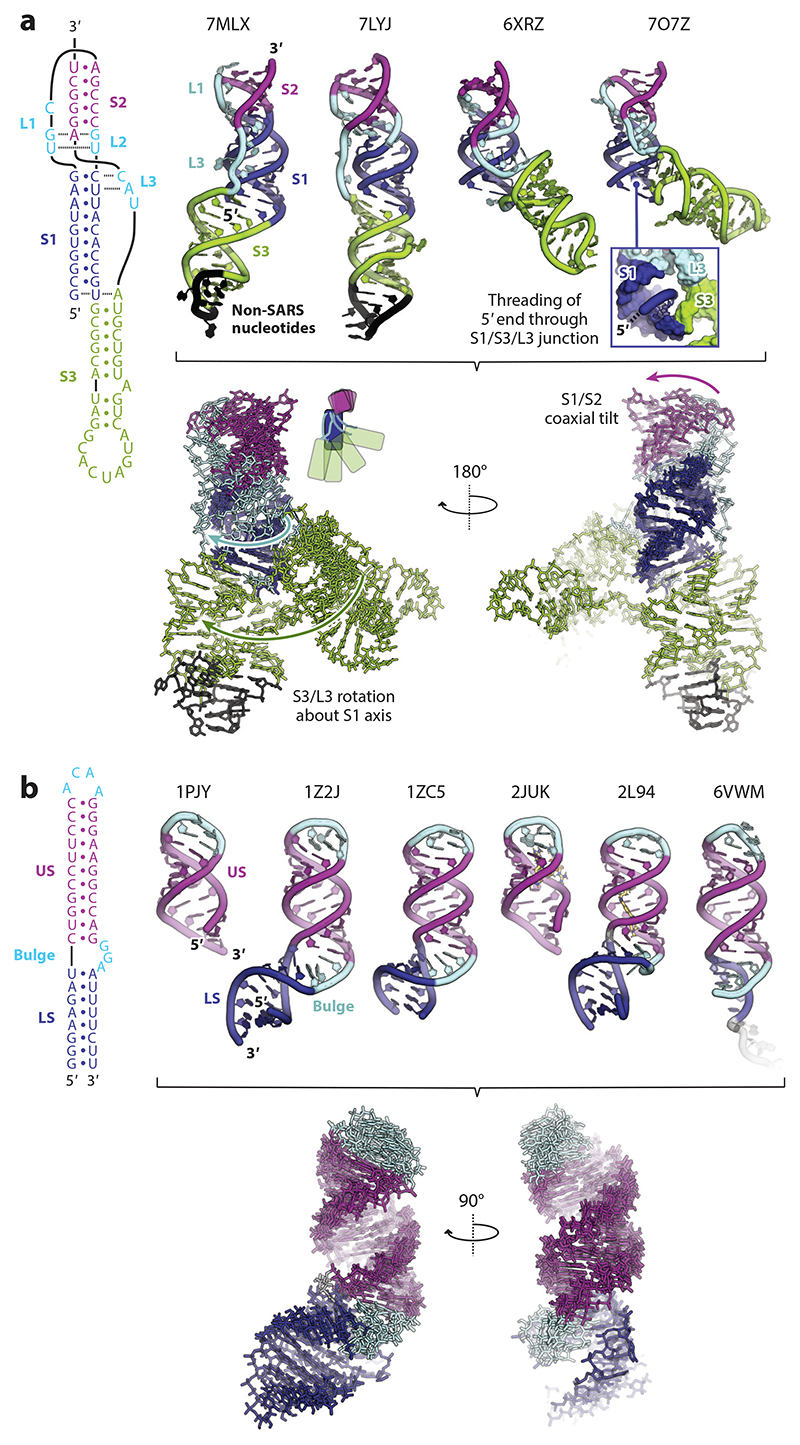
Illustrating the conformational repertoire of two −1PRF stimulatory elements. (*a*) Topological diagram and structures of SARS-CoV-2 pseudoknots, color coded by feature. PDB codes are indicated above each structure. (*inset*) Example of 5′ end threading through the S1/S3/L3 junction. Backbone alignment by least-squares superposition reveals the principal modes of flexibility. (*b*) As in panel *a* but for HIV-1 stem-loops. Abbreviations: −1PRF, programmed −1 ribosomal frameshifting; HIV-1, human immunodeficiency virus 1; PDB, Protein Data Bank; SARS, severe acute respiratory syndrome; SARS-CoV-2, severe acute respiratory syndrome coronavirus 2.

Recently, the structure of the SARS-CoV-2 frameshift-stimulatory pseudoknot has been solved alone by cryo–electron microscopy (cryo-EM) (50) and X-ray crystallography (51, 52), and in the context of a stalled-ribosome complex (53) (Figure 4*a*). Previous analyses of this class of pseudoknot, typified by possession of a long S1 (of ~11 bp) and long L3 (~30 nucleotides), had suggested that coronavirus pseudoknots might lack some of the complex conformational features identified in other stimulatory pseudoknots at that time (30, 54–59). For example, loop-helix interactions were considered unlikely as L3 of the IBV pseudoknot could be shortened to the minimum length required to span S1 (8 nucleotides), and the base composition of this short loop flipped, without affecting −1PRF (54). Further, interstem kinking through an unpaired residue at the S1/S2 junction was also considered unlikely as the terminal base pair of the IBV PK S1 (UG) could be functionally replaced by a more stable GC or CG pair (54). Indeed, the predominant aspect, beyond pseudoknot formation, was a requirement to maintain an S1 of at least 11 bp, with longer stems (up to at least 13 bp) tolerated (56). In examining the four recent SARS-CoV-2 structures, a standout feature is the presence of three helices, the traditional stems (S1 and S2) and a third stem (S3) formed within (and composing most of) L3. The relative position of S3 varies in the four structures, highlighting the conformational plasticity of this element (Figure 4*a*). It is stacked vertically onto the S1/S2 helices in the crystal structures (51, 52) but angled away from the coaxial S1/S2 stack in the cryo-EM reconstructions (50, 53). The S1/S2 axis also varies, with minor coaxial tilting between the four structures. It is uncertain whether the differences in L3 and S3 orientation are related to function, and to what extent the linear, vertically stacked conformations of S3 are related to crystal packing (51, 52). However, S3 in this configuration would likely clash with the ribosomal helicase at the 5′ end of the pseudoknot, so a movement of S3 and L3 toward more angled conformations may be required on ribosome approach. In the context of the ribosome (53) S3 does not make any obvious contacts with the small subunit, and in the closely related SARS-CoV pseudoknot, L3 can largely be deleted with only modest effects [albeit with certain caveats (59, 60)]. Another noticeable feature is that in three of the structures, the single-stranded 5′ end of the RNA threads through a ring formed by the S1/S3 junction and S3-L3 (Figure 4*a*), which may have implications for unwinding. From molecular dynamics, Yan and coworkers (61) propose that the straight (X-ray) and angled (cryo-EM) structures represent steps on a folding pathway, with an angled L3 formed upon threading of the 5′ end of the mRNA. However, the Roman et al. (52) structure is threaded but has a linear L3, so this relationship needs further assessment. The 1.3-Å structure of Jones & Ferré-D’Amaré (51) reveals that the core of the SARS-CoV-2 pseudoknot is formed by a stack of four consecutive base triples, in which nucleotides in S1, S2, L1, L2, and L3 are plaited together. Three of these are loop-helix contacts, but the fourth represents an interaction between individual nucleotides from each of the three loops, bringing them together and extending stacking between S1 and S2. Overall, this set of unique base-triple interactions generates a highly structured, continuously stacked connection between the S1 and S2 helices. From these analyses, it is clear that the long S1 class of pseudoknot also has complex structural features.

#### 2.2.2 Stem-loops

A variety of animal and plant virus −1PRF signals have stem-loop stimulators, including human astrovirus serotype-1 *1a/1b* (HAst-1), Sindbis virus (SINV) *6K/TF* (62), HTLV-II *gag/pro*, HIV-1 *gag/pol*, Cocksfoot mottle sobemovirus *2a/2b*, and red clover necrotic mosaic virus *p27/p57* (reviewed in 58). Mutational analysis, RNA secondary structure probing, and phylogenetic sequence comparisons indicate a strong likelihood that these elements act discretely and are not part of a higher order structure, such as a pseudoknot or kissing complex. Presently, it is not clear whether they have structural features that might account for their capacity to promote −1PRF. The HAst-1 stimulatory RNA, for example, has only a small, 7-bp G-C-rich stem and a 10-nucleotide loop without obvious stabilizing motifs, and one would not expect it to pose a problem for the 80S ribosome (63). Structural information is available only for the HIV-1 stem-loop, through solution NMR (64–67) (Figure 4*b*). This revealed a two-stem structure in which a three-purine (GGA) bulge separates a highly stable upper stem capped by an ACAA tetraloop from a lower stem formed by pairing of the spacer region with a complementary sequence 3′ of the upper stem. Base stacking in the bulge forms a wedge, introducing a 60° bend between the helices, reminiscent of the kink described for the MMTV *gag/pro* pseudoknot and related elements. The relevance of these structural features to −1PRF is uncertain, and compared to the conformational repertoire of the SARS-CoV-2 element (Figure 4*a*) the HIV-1 element appears relatively invariant (Figure 4*b*). However, the lower stem must denature to allow the ribosome access to the slippery site; thus, it could alter translation kinetics as the ribosome approaches the slippery sequence or act as a positioning element to allow the upper stem to interact productively with the ribosome (65, 67). Importantly, stem-loop stimulators produce levels of frameshifting far in excess of slippery sequences alone (often 5–10% of ribosomes change frame) and can be tweaked to even higher levels. Stabilizing the bottom of the HIV-1 upper helix with GC pairs doubles the wild-type efficiency [to 20% (68)], and stem-loops engineered in the context of the SRV-1 *gag/pro* signal can also induce high-level frameshifting, in a length-dependent manner (69). Given the paucity of structural information, we cannot exclude that stem-loop stimulators contain unanticipated features that might contribute to function. Alternatively (as discussed in Section 2.3), stimulatory elements beyond the mRNA may contribute, for example, through the action of *trans-*acting proteins or nascent peptide effects. Bao and colleagues (70) recently proposed that stem-loop stimulators function by forming inhibitory interactions with the ribosomal A-site, dependent upon a specific stem length and necessitating the presence of the loop (discussed in Section 3.3).

#### 2.2.3 RNA mechanical stability versus conformational plasticity

Many features of stimulatory pseudoknots have been linked rationally to their capacity to induce frameshifting, including complex geometry, a minimum thermodynamic stability of the stems, appropriate stem length, optimal stability of the first few base pairs of S1, and even the G-richness of the 5′ arm (56; reviewed in 45, 58). Another potential correlate of PRF efficiency is the mechanical stability of the pseudoknot, as judged by single-molecule force spectroscopy using optical tweezers, with −1PRF positively correlated with the cooperative one-step unfolding force in many, but not all, studies (49, 71–74). A competing idea is that instead of mechanical stability, RNA conformational plasticity determines −1PRF efficiency at some signals (72, 75–80). In these studies, plasticity is related to the apparent number of conformations that any given RNA can adopt, often inferred by the presence of multiple unwinding events at discrete forces, or the broadness of the force distribution at which unwinding occurs. A striking example comes from the analysis of pseudoknot dynamics in the flavivirus West Nile virus *NS/NS*′ −1PRF signal (81). This highly efficient frameshift stimulator [up to 70% (76)] has complex, heterogeneous dynamics, with two parallel pathways each containing multiple intermediates. Remarkably, the occupancy of pseudoknotted conformations appears to be too low for static pseudoknots to account for the high levels of −1PRF measured. Halma and colleagues (81) propose that efficient −1PRF is linked to the frequency at which the stimulator changes conformation when under tension from ribosomal attempts to translocate through it. Indeed, measurements of the force-induced unfolding of stimulatory RNAs reveal a correlation between −1PRF and the Shannon entropy of state formation—essentially a measure of the uncertainty over which RNA conformation is formed (79). Conformational plasticity could modulate helicase activity by offering alternative substrates and lead to abrupt tension fluctuations that may be transmitted to the tRNA-mRNA complex. Conformational heterogeneity has also been observed in the SARS-CoV-2 frameshift element (61, 82–86).

Structural variation in stimulatory RNAs can also be induced through longer range interactions with elements outside of the core motif and can be a prerequisite for function. The barley yellow dwarf virus (BYDV) *orf 1/orf 2* signal has a local stem-loop stimulatory element (87), but efficient frameshifting to produce the viral replicase requires the formation of a long-range (4 kb) pseudoknotting interaction with the 3′ end of the viral genome (88). This long-range interaction is part of an elegant strategy to balance use of the genomic RNA template for translation or replication (89). Recent studies of SARS-CoV-2 have also unearthed longer range interactions involving the frameshift element that appear unnecessary for activity in vitro but may impinge on function in vivo (90–92).

### 2.3 Proteins and Peptides

#### 2.3.1 Protein stimulators

Identification of the first viral protein stimulator of frameshifting evolved from the work of Fang and colleagues (93) who identified a novel PRF signal in the arterivirus porcine reproductive and respiratory syndrome virus (PRRSV). The PRRSV genome, a ~15-kb positive-sense RNA molecule, harbors two PRF signals (Figure 3*e*). A canonical RNA pseudoknot-dependent −1PRF signal is located at the junction of 1a and 1b ORFs, and a second signal, which stimulates both −2PRF and −1PRF, is located within the region of ORF1a that encodes a multifunctional replicase subunit, nonstructural protein 2 (nsp2). Here, about 20% of ribosomes translating nsp2 frameshift into the −2 reading frame to generate a fusion protein (nsp2TF) and 7% into the −1 frame generating a truncated form of nsp2 (nspN). The RNA downstream of the AG_GUU_UUU slippery sequence employed for −2/−1PRF does not harbor an RNA structure, but mutations within a phylogenetically conserved CCCANCUCC motif located 10 nucleotides downstream of the shift site reduce or inhibit frameshifting, consistent with the presence of a 3′ stimulatory element of some form. Subsequently, an essential role for the viral replicase subunit nsp1β was identified, as a *trans*-activator of both −2 and −1PRF (94) when in complex with the cellular poly(C) binding protein (PCBP) (95). The nsp1β/PCBP complex binds to the C-rich motif downstream of the slippery sequence and appears to mimic a structured RNA stimulator of programmed frameshifting. Little is known about the molecular basis of frameshifting at this site, but biochemical analysis, including small-angle X-ray scattering (SAXS), indicates that the proteins associate with the C-rich motif in a 1:1:1 complex and that both nsp1β and PCBP associate directly with the mRNA (96) (Figure 3*e*). Nsp1β can functionally interact with each of three PCBP paralogs with some variation in the consequent nsp2:nspTF:nspN ratio, perhaps offering some useful flexibility in gene expression (95).

A second protein-stimulated PRF signal derives from the work of Loughran and colleagues (97), who identified highly efficient (~70%) −1PRF signals in encephalomyocarditis virus (EMCV) (Figure 3*f*) and Theiler’s murine encephalomyelitis virus (TMEV). Frameshifting occurs at a conserved G_GUU_UUU sequence within the 2B-encoding region, generating a novel protein 2B*, but critically, it is dependent upon virus infection. In both viruses, a predicted RNA stem-loop structure is present that could act as a stimulatory RNA but is located too far 3′ of the slippery sequence to interact with the ribosome (spacer is 13–14 nucleotides). Subsequently, protein 2A was identified as the viral cofactor, binding to the stem-loop and forming a protein-RNA complex that is highly active in PRF stimulation (98, 99). A feature of the nsp2/2B* signal is that it allows temporal regulation of virus gene expression. Early in infection, the absence of 2A permits uninterrupted translation of 3′-encoded replication proteins. Later in infection, cytoplasmic 2A accumulates, stimulating PRF and diverting ribosomes from the polyprotein into the 2B* ORF and downregulating replication protein synthesis. Frameshifting thus provides an elegant and economic way to build up replication capacity early in infection, while providing a translational bias that favors virion production at late time points. The X-ray crystal structures of 2A from EMCV and TMEV have been solved, revealing a novel RNA binding fold termed a beta-shell (74, 100) (Figure 3*f*). Study of the dynamics of the RNA element to which this protein binds, by single-molecule optical tweezers, suggests that the RNA can adopt stem-loop and pseudoknot-like organizations and that 2A selectively binds to and stabilizes the latter.

#### 2.3.2 Protein inhibitors

Recently, Wang and colleagues (101) identified an interferon (IFN)-stimulated gene (ISG) product, Shiftless (SHFL), encoded by *C19orf66*, that restricts HIV-1 replication through downregulation of *gag/pol* frameshifting. It was the first identified protein that acts as a repressor of −1PRF and the first example of an ISG that targets a translational recoding process. SHFL appears to be a broad-spectrum inhibitor of frameshifting, typically reducing the measured efficiency by 0.5 to fourfold, a magnitude likely sufficient to suppress replication of a variety of viruses employing PRF. The protein has RNA and ribosome binding activity (101, 102), consistent with its role, but no obvious specificity for stimulatory RNAs (102). The inhibitory mechanism remains uncertain but may involve premature removal of ribosomes from the mRNA at the frameshift site (101). Notably, SHFL has other antiviral roles not obviously linked to PRF and has alternatively been named RyDEN, C19orf66, and IRAV (reviewed in 103).

Zimmer and colleagues (104) have described another inhibitor of −1PRF, the short isoform of the zinc-finger antiviral protein. Like SHFL, this is an IFN-induced protein that can associate with ribosomes, but its activity is specific to the SARS-CoV-2 signal (and the closely similar −1PRF signal of SARS-CoV), binding directly and with specificity to the pseudoknot-stimulatory element and interfering with its refolding.

#### 2.2.3 Nascent chain effects

There is growing evidence that the amino acid sequence and conformational state of the nascent polypeptide can modulate −1PRF, via mechanical force transmitted through peptidyl tRNA. In SINV, the efficiency of the *6K/TF* signal is modulated by the frequency at which a transmembrane domain (TM2) within the zero frame E2 protein undergoes translocon-mediated membrane integration, with the spacing between the C-terminal residue of TM2 and the slippery sequence critical to this activity (105). Saturation mutagenesis of sequences upstream of the slippery sequence has revealed that −1PRF is sensitive to a variety of mutations that alter the amino acid sequence within the portion of the nascent chain that has been translated at the point of frameshifting, including the TM2 domain, which may reduce the force on the nascent chain or delay its transmission until after the ribosome is beyond the slippery sequence. Additionally, certain residues within the exit tunnel and between exit tunnel and translocon have also been implicated (106). Here, the effect on −1PRF may reflect effects on translocon-mediated cotranslational folding. Alternatively, it may be linked to hydrophobic interactions with the exit tunnel [as also seen with SARS-CoV-2 (53)] or slow decoding of certain codons, in each case altering translation kinetics.

## 3 Mechanistic Aspects of Ribosomal Frameshifting

### 3.1 Ribosomal Pausing

Almost since the first description of −1PRF (6), the idea that stimulatory RNAs would pause the ribosome to give more time for tRNA realignment was pervasive. Early pausing assays included edeine-synchronized in vitro translation time courses (107) and ribosome heel printing (108–110). The latter technique exploits the capacity of the ribosome to protect ~30 nucleotides of mRNA from external ribonuclease addition (111); the ribosome-protected fragments (RPFs) are annealed to a complementary single-stranded DNA template and the 5′ end localized by primer extension. Together, these techniques established that stimulatory RNAs and proteins do indeed pause ribosomes, with translation intermediates visible over periods of minutes in edeine assays, and that the site of pausing is localized over the slippery sequence. Further, pausing was enhanced upon inactivation of the slippery sequence, the first indication that frameshifting might give the ribosome a route of escape from a kinetic trap. Conjectures drawn from these assays, however, must be viewed in context as they do not report on steps in the elongation cycle (nor on individual ribosomes), which are typically measured in tenths of a second (112). More recently, ribosomal profiling has been exploited to examine −1PRF and ribosomal pausing in virus-infected cells. Appositely, profiling is an elaboration of the heel printing assay, where the RPFs (the reads) are instead mapped by deep sequencing and aligning to host and viral genomes (113). With certain caveats (16), profiling permits the relatively facile measurement of −1PRF efficiencies in vivo through quantitation of read coverage in the relevant ORFs. A recent discovery from this approach is that −1PRF at the PRRSV 1a/1b signal increases over a time course of infection, contrary to the belief that these sites generally function to deliver a fixed ratio of gene products through the life cycle (114). This may have implications for many other viruses using this mechanism of gene expression. Interestingly, at RNA-structure-dependent sites of −1PRF, no substantial RPF peaks accumulate that would correspond to ribosomes stalled over the slippery sequence, indicating that any induced pause in vivo occurs too rapidly to be detected by this methodology. However, at both the 2A-dependent signal of EMCV (98) and the PRRSV nsp1β/PCBP signal (114), significant peaks are seen, indicating a longer pause at the protein-dependent −1PRF signals. At the 2A-dependent site, pausing is also enhanced substantially by inactivation of the slippery sequence, supporting the earlier view that frameshifting may provide an exit pathway for elongation-stalled ribosomes.

### 3.2 −1PRF in the Context of Translocation

**Figure 5:**
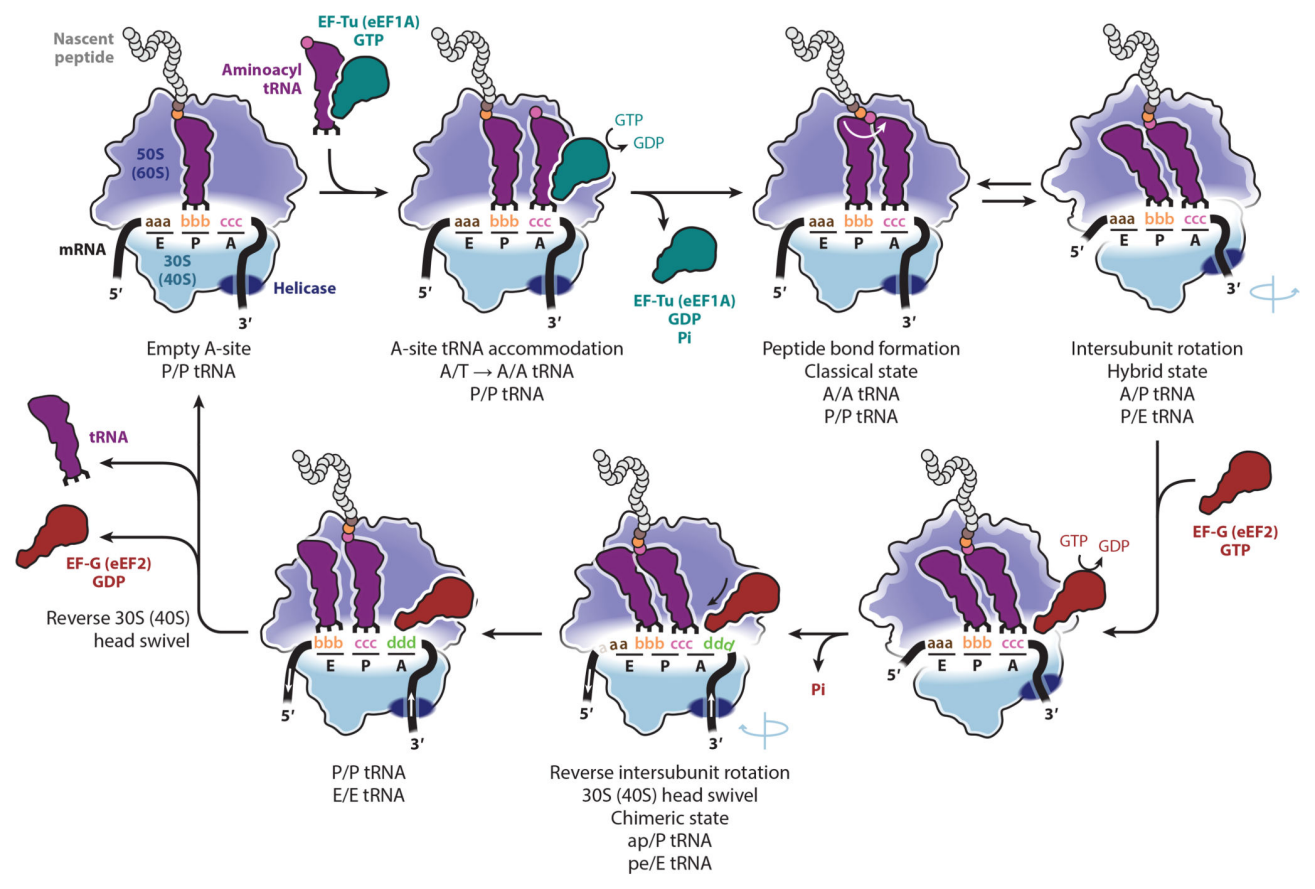
Schematic of the elongation cycle of translation. The steps and intermediates shown are based on prokaryotic translation, with equivalent eukaryotic factors shown in brackets. Abbreviations: A, aminoacyl site; E, exit site; eEF1A, eukaryotic elongation factor 1A; eEF2, eukaryotic elongation factor 2; EF-G, elongation factor G; EF-Tu, elongation factor Tu; P, peptidyl site; T, EF-Tu-/eEF1A-tRNA binding site.

It is appropriate to consider −1PRF within the context of the (prokaryotic) ribosomal elongation cycle, particularly the step of translocation, now generally considered to be the stage where frameshifting takes place (5, 157) (Figure 5).

At this point, peptidyl-tRNA is in the P-site with a newly accommodated tRNA in the A-site, the so-called classical state. Almost immediately, peptide bond formation takes place, transferring the nascent peptide to the A-site tRNA and unlocking the ribosome. This permits the ribosomal subunits to spontaneously and reversibly rotate with respect to each other, with forward rotation of the small subunit body and partial rotation of the head domain coupled with movements of tRNAs such that the acceptor stems of the P- and A-site tRNAs move to the subsequent [exit (E) and P] sites of the 50S subunit but the anticodon stem-loops remain in place on the 30S subunit. This P/E A/P state hybrid state ribosome can spontaneously fluctuate with the classical state, but upon elongation factor G (EF-G; eEF2 in eukaryotes) binding to the hybrid state, reverse rotation of the 30S subunit body takes place, as well as full forward head rotation, generating a conformation in which the tRNAs occupy chimeric ap/P pe/E positions, where the anticodon stem-loops of the tRNAs occupy a unique binding site on the small ribosomal subunit partway between the classical and hybrid positions. Subsequently, the rate-limiting reverse rotation of the 30S head domain and EF-G release allows the tRNAs to move into their canonical P/P E/E binding states, leaving the A-site vacant and ready for the next round of elongation to commence.

The effect of frameshift-stimulatory RNAs on the elongation cycle has been studied almost exclusively in reconstituted prokaryotic translation systems, using naturally occurring (prokaryotic cellular) −1PRF signals such as *dnaX* and IS3 (often with an additional 5′ stimulatory Shine-Dalgarno motif), or hybrid signals, with the IBV or HIV stimulatory element, coupled with an optimized prokaryotic slippery sequence (e.g., A_AAA_AAG). Overwhelmingly, stimulatory RNAs have been shown to slow translocation, impairing aspects of movement, particularly reverse rotation of the head domain, with persistence of classical-hybrid state fluctuation and the slow release of EF-G (21, 112, 115–119). In the model of Caliskan and coworkers (112), the pseudoknot acts to inhibit backward rotation of the 30S head, leading to EF-G retention. In the absence of a slippery sequence, the progression of the ribosome is stalled dramatically and EF-G release is extremely slow, consistent with the results of pausing assays discussed earlier. The slippery sequence is posited to allow the ribosome to change its position with respect to the pseudoknot, facilitating more rapid completion of translocation and favoring −1PRF kinetically. In some studies, the presence of the stimulatory RNA leads to ribosomes attempting multiple EF-G-driven translocation attempts as they strive to overcome the barrier (21, 115, 120). Such distressed EF-G activity is symptomatic of a frameshift-prone environment, where failure to complete translocation rapidly is associated with loss of reading frame (121). Indeed, there is growing evidence that mutations within EF-G domain IV, which contacts the A-site tRNA anticodon, can lead to dramatically increased −1 frameshifting (119).

During a pause in the rotated state, repositioning on the slippery sequence might be driven by thermodynamics (20, 21), but we suggest that tension also develops, pulling the mRNA and biasing direction. Varying the length of the spacer separating a U_6_A heptamer from a frameshift-stimulatory stem-loop, pseudoknot or base-paired oligonucleotide alters the proportion of ribosomes entering the −1 or −2 reading frame (122). In all cases, −2PRF is optimal at a spacer length 1–2 nucleotides shorter than that optimal for −1 frameshifting. The shorter spacer is believed to increase tension on the mRNA such that when the tRNA detaches, it more readily enters the −2 frame on the slippery sequence. Tension may also be developed in other ways, for example, if stimulatory RNAs sample alternative conformations during the pause, or through nascent peptide interactions with the exit tunnel or membranes.

### 3.3 Structural Studies of Ribosome Complexes

Recently, structural studies of ribosomes engaged with stimulatory elements have been published that offer new insights into the frameshift process.

#### 3.3.1 Ribosomes paused at −1PRF signals

**Figure 6:**
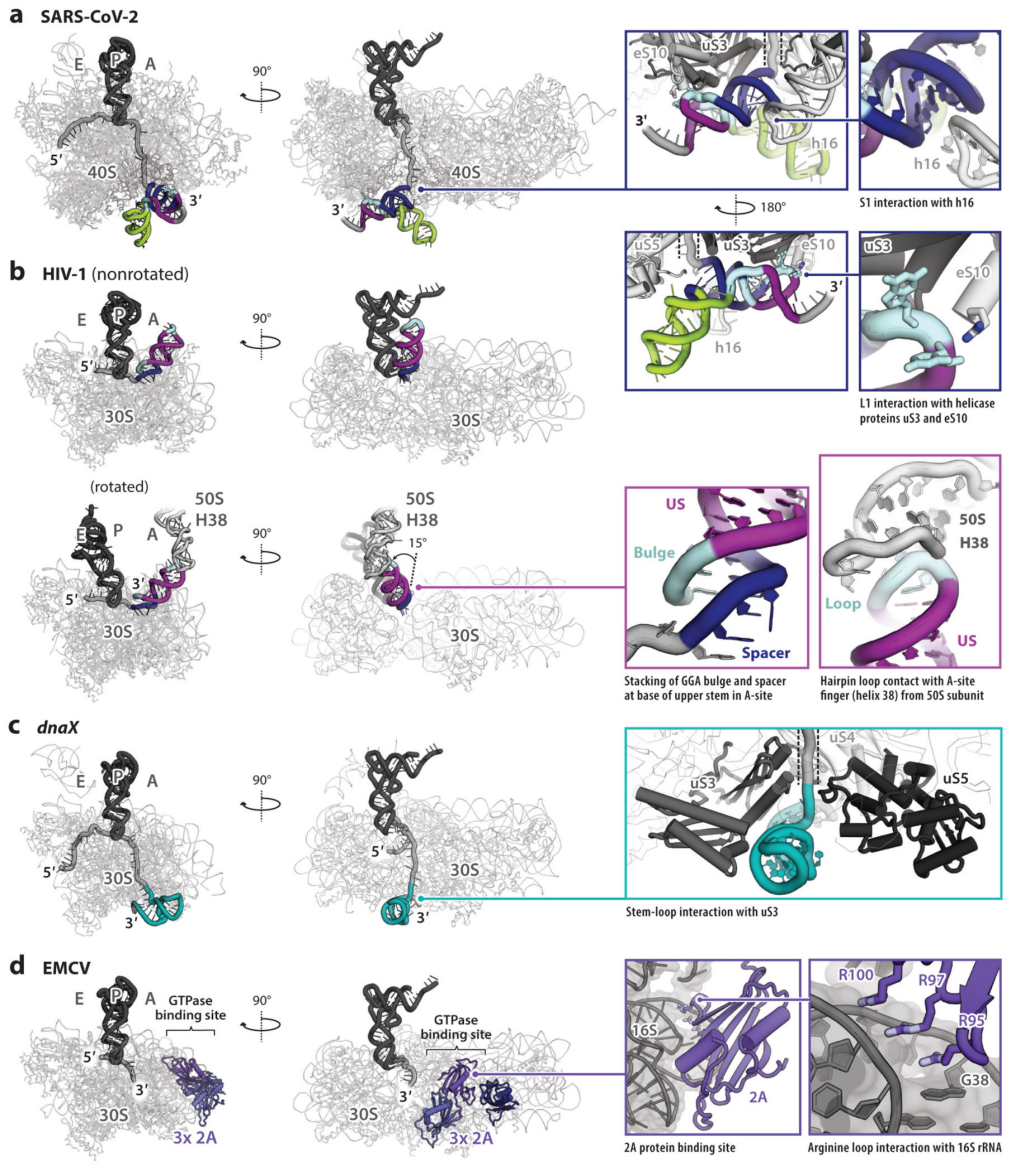
Interactions between the ribosome and −1PRF stimulatory elements. The large ribosomal subunit is omitted for clarity. (*a*) 80S ribosomes stalled one codon before the slippery sequence in a preframeshift complex with the SARS-CoV-2 pseudoknot. The P-site tRNA (*dark gray*), mRNA (*light gray*), and pseudoknot (colored as in previous figures) are shown. (*inset*) Zoomed-in views showing interactions between the SARS-CoV-2 pseudoknot, ribosome helicase subunits uS3 and eS10, and 18S rRNA helix 16. (*b*) Reconstituted *Escherichia coli* 70S ribosomes at the HIV-1 frameshift site. The stem-loop can fold within the A-site, preventing tRNA delivery. (*inset*) Spacer and GGA bulge stack against base of upper stem in the A-site. In the P/E rotated state, the stem-loop rotates by ~15° and contacts helix 38 from the large subunit. (*c*) Reconstituted *Thermus thermophilus* 70S ribosomes at the bacterial *dnaX* frameshift site. The stem-loop stimulatory element is visible at the ribosome helicase. (*inset*) The stem-loop is in close contact with ribosome helicase subunit uS3. (*d*) Reconstituted *E. coli* 70S ribosomes in complex with EMCV 2A. (*inset*) 2A binds to 16S rRNA at the GTPase binding site, potentially interfering with tRNA delivery and/or translocation. Abbreviations: 1PRF, programmed −1 ribosomal frameshifting; A, aminoacyl-site; E, exit-site; EMCV, encephalomyocarditis virus; HIV-1, human immunodeficiency virus 1; mRNA, messenger RNA; P, peptidyl-site; rRNA, ribosomal RNA; SARS-CoV-2, severe acute respiratory syndrome coronavirus; tRNA, transfer RNA.

In two studies, two high-resolution structures of ribosomes paused at −1PRF signals are available, 80S RRL ribosomes translating an ~800-nucleotide portion of the SARS-CoV-2 genome, encoding the C terminus of nsP10, nsP11 and the N terminus of nsP12 (53), and *Escherichia coli* 70S ribosomes assembled on a short HIV-1 *gag/pol* RNA (67). In neither study (53, 67) was a functional slippery sequence present. In the SARS-CoV-2 investigation, stalled 80S complexes were purified by virtue of an N-terminal FLAG-tag on the nascent peptide. To maximize the proportion of ribosomes paused at the −1PRF signal, the authors replaced the second codon of the slippery sequence (uppercase) with a translation termination codon (italicized; uuU_UUA_*UAA*) and flooded the RRL with a mutant eukaryotic release factor [eRF1 (AAQ)] unable to release the nascent polypeptide. Two key ribosome complexes were solved with implications for −1PRF mechanisms. The major, pseudoknot-containing complex, comprising 70% of the ~1.3 million particles classified, was an unrotated ribosome with P- and E-site tRNAs bound conventionally to the mRNA in the zero frame. Curiously, the ribosome has stalled one codon upstream of its expected position, decoding uuU (Phe) in the P-site with A-site (UUA) empty and no eRF1 (AAQ) on the complex, presumably as the stop codon has not yet entered the decoding site. This structure can thus be viewed as a preframeshift state. A second, lower-abundance complex (~4% of particles), also unrotated, has fully engaged the slippery sequence, with leucine tRNA decoding UUA in the P-site and the A-site (UAA) occupied by eRF1 (AAQ) as expected, itself complexed with ABCE1, a factor associated with translation termination and recycling of ribosomes (123). In this termination complex, the pseudoknot appears to be absent, having been unwound or become disordered. The preframeshift complex reveals both ribosome and pseudoknot in exquisite detail and provides some interesting insights into the interactions that occur on the stalled complex (Figure 6*a*). Consistent with potential unwinding by the intrinsic ribosomal helicase (35, 38), the pseudoknot is wedged close to the mRNA entry channel, with nucleotides in the spacer region close to S1 interacting with basic residues in the C-terminal domain of ribosomal proteins associated with uS3 and to some extent eS30. L1 also contacts uS3, as well as the C-terminal tail of eS10. A direct interaction between 18S ribosomal RNA (rRNA) helix 16 and S1 may restrict head and body rotations during translocation, promoting −1PRF (Figure 6*a*). Nevertheless, for the slippery sequence to be correctly positioned within the decoding center, the ribosome must advance three nucleotides from its observed position, likely unwinding (at least) the base of S1. It is unclear to what extent the pseudoknot would remain folded or the L1-uS3-eS10 contacts would persist in such a complex. Therefore, the details associated with a bona fide pause at the slippery sequence remain elusive.

An earlier reconstruction of 80S ribosomes paused at the IBV signal at lower resolution (13–20 Å cf. 2.4–7 Å) revealed features consistent with stalling during translocation, with eEF2 bound and a single, distorted, hybrid-state peptidyl-tRNA (124). Density assigned as the pseudoknot was visible at the mRNA entry channel. A mechanical model of frameshifting was proposed in which the pseudoknot resists unwinding by the helicase, compromising translocation by putting tension on the mRNA, leading to bending of the tRNA anticodon and, ultimately, repositioning of the tRNA on the slippery sequence in the −1 reading frame. While the model is not dissimilar to those formulated from more recent work, this earlier structure is clearly different, and perhaps artifactual, potentially arising from the difficulties in accurately fitting atomic components into low-resolution cryo-EM maps or unsuspected contamination by the recently described inactive ribosomes present in RRL (125).

In the 70S-HIV cryo-EM study (67), two predominant 70S classes were identified that differed in P-site tRNA position (P/P or P/E), but in either case, the A-site was occupied, remarkably, by the HIV stem-loop stimulatory RNA (Figure 6*b*). The base of the upper stem stacks against the 5′ spacer and purine-rich bulge nucleotides, which adopt a helical conformation, also in the A-site. In the rotated P/E state, the helical axis of the upper stem is tilted by approximately 15°, establishing a direct contact between the loop and the A-site finger comprising helix 38 of the large subunit. These observations may explain the strict requirements for stem length and are consistent with tRNA-binding experiments that indicate that the presence of the HIV-1 stem-loop reduces A-site tRNA binding to ribosomes (70). Those researchers hypothesize that mRNA stem-loops can transiently escape the 70S helicase and dock into the A-site to compromise translation, and that frameshifting in this case may occur by an alternate P-site-only slippage model (67). They also demonstrate impaired tRNA binding to the A-site at the bacterial *dnaX* signal, which employs a stimulatory stem-loop. However, a cryo-EM structure of 70S ribosomes assembled on a similar *dnaX* mRNA showed that the A-site was empty and the stem-loop was observed in the expected position at the ribosome helicase (126) (Figure 6*c*). It is possible that the two states may interconvert, as displacement of the stem-loop from the A-site is a prerequisite for translation to continue in either frame. Future work will test whether the method of assembly of such complexes influences the likelihood that the stem-loop will enter the A-site.

#### 3.3.2 Ribosomes in complex with *trans*-acting factors

The EMCV 2A protein stimulates −1PRF by stabilizing a pseudoknot-like conformation of the RNA stimulatory element, as discussed above (Section 2.3). However, 2A also binds to ribosomes. A cryo-EM structure of a 2A-70S complex showed that 2A interacts with 16S rRNA at the factor binding site of the small subunit, potentially competing with translational GTPases (74) (Figure 6*d*). This may slow down translocation or tRNA delivery during −1PRF, but the mechanistic importance of this requires further evaluation because 2A also has other roles, including the inhibition of cap-dependent initiation on host mRNAs (reviewed in 99).

## 4 Targeting −1PRF for Antiviral Intervention

**Figure 7:**
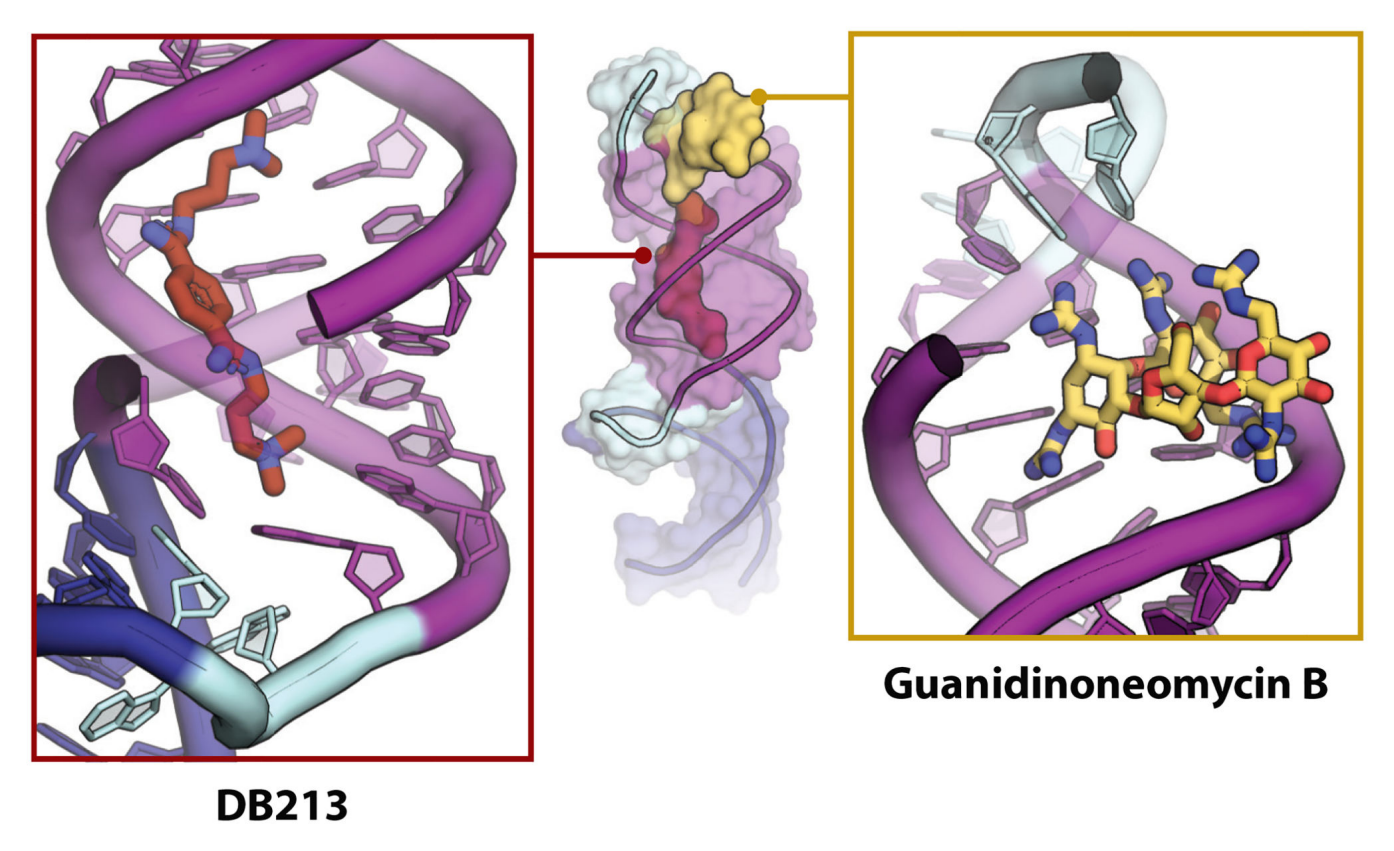
Small-molecule binding sites on the HIV-1 stem-loop, targeting the upper helix. Compounds are shown as sticks in the zoomed-in views. Abbreviation: HIV-1, human immunodeficiency virus 1.

The vital role of −1PRF in the replication cycles of numerous virus pathogens has prompted the search for antiviral agents targeting the process. Early studies revealed that certain antibiotics targeting ribosomes (e.g., anisomycin, sparsomycin, and cycloheximide) could modulate L-A virus frameshifting and replication (11, 127), but their specificity and activity against other viruses have not been rigorously tested [although the aminoglycoside geneticin is active against SARS-CoV-2 (128)]. Concurrent with these studies, high-throughput screening identified a candidate antiframeshift drug DB213 (also known as RG501) that binds the HIV-1 stem-loop, stimulates frameshifting at the HIV-1 signal about twofold, and inhibits HIV-1 replication in tissue culture, but the specificity of the effect was complicated by cytotoxicity (129, 130). The drug also stimulates frameshifting at the stem-loop-containing signals of HIV-2, simian immunodeficiency virus 1, and HTLV-1 *gag/pro*, but not HTLV-1 *pro/pol*, which contains a pseudoknot (131). DB213 is now known to bind to the HIV stem-loop major groove through electrostatic interactions, stabilizing the structure and increasing −1PRF by an unspecified route (66) (Figure 7). The binding mode of DB213 is relatively nonspecific; it may bind to other RNAs with a long duplex and could conceivably also inhibit −1PRF through binding to rRNA. The HIV stem-loop also binds the aminoglycoside antibiotic guanidinoneomycin B (GNB), through an interaction with the major groove, which is broadened (132) (Figure 7). GNB stabilizes the helix and slightly repositions the ACAA tetraloop. The compound has not been tested in −1PRF assays and RNA binding specificity is a concern, but it blocks HIV-1 replication modestly (133). To improve affinity and specificity, Marcheschi and colleagues (66) propose that DB213 (or a derivative) might be covalently linked to GNB, or to compounds that target the GGA bulge, such as doxorubicin (134). Miller and colleagues (135) have since developed thematically related compounds that target the HIV stimulatory RNA with increased affinity, stimulating frameshifting and reducing HIV-1 replication with only modest cytotoxicity (136), although there have been no clinical trials as yet. The advent of SARS-CoV and SARS-CoV-2 has increased focus on the development of strategies targeting −1PRF (reviewed in 137–140). A small-molecule inhibitor of SARS-CoV −1PRF (compound 43/MTDB) identified by Park and colleagues (141) is also active against the SARS-CoV-2 signal (142) and natural variants (143), perhaps by reducing pseudoknot plasticity (144). In one study, MTDB did not affect −1PRF but had a significant effect on SARS-CoV-2 replication, questioning the specificity of the molecule (53). However, certain fluoroquinolones (merafloxacin and KCB261770) identified in unbiased high-throughput screens robustly inhibit −1PRF in a range of betacoronaviruses and impede SARS-CoV-2 replication, although the mechanism is not known (53, 145, 146). Munshi and colleagues (147) have also examined the effect of several compounds on human and bat coronavirus −1PRF signals. Oligonucleotide targeting of frameshift signals may also permit antiviral intervention, by destabilization, stabilization, or cleavage of stimulatory RNAs, and potentially through decreasing conformational heterogeneity, but there are few published studies to date (139). Neuman and colleagues (148) deployed a peptide-conjugated, phosphorodiamidate morpholino antisense oligomer against the SARS-CoV pseudoknot stems with replication-inhibitory effect, and Zhang and colleagues (50) used locked nucleic acid oligonucleotides to target the SARS-CoV-2 pseudoknot. The inhibitory effect in each study was modest (~1–2 log) but promising.

As with all antivirals, the specificity, activity and toxicity of treatments must be considered alongside the possibility of escape mutations. Frameshift-modulatory drugs would potentially also affect any cellular frameshifting signals, but in the case of an acute virus infection, it is hoped that there would not be a prolonged detrimental effect over a short course of treatment of an infected patient.

## 5 Concluding Remarks and Future Directions

So where do we stand in the study of the how, why, when, and where of PRF [to paraphrase an early review (149)]? A model of −1PRF is emerging where stimulatory elements, through resistance to unwinding, mechanical force, conformational plasticity, and direct interactions with the ribosome, compromise translocation and eEF2 function, slowing the process and potentially straining the mRNA, facilitating movement into the −1 frame on the slippery sequence. At first glance, some features of this model appear counterintuitive or even contradictory. How can resistance to mechanical unwinding and conformational plasticity both drive frameshifting? Why is there not an overall correlation between −1PRF efficiency at different signals and stability of the stimulatory element? These conflicts can be resolved if one accepts that a general mechanism of −1PRF is an oversimplification. As the outcome is the same in each case, i.e., the ribosome moves backward by one nucleotide, it is tempting to invoke the reductionist approach of mechanistic universality when thinking about the process. But why should one universal mechanism exist? The structural diversity of stimulatory elements reflects the vast evolutionary distance between groups of viruses exploiting −1PRF, and it is possible that the convergent evolution of multiple mechanisms may have arrived at the same result. Models of frameshifting would therefore benefit from high-resolution structural and kinetic analyses of a wider variety of stimulatory elements, including structures of elongating ribosomes decoding a slippery sequence. Single-molecule and ensemble kinetic analyses of 80S ribosomes engaged with a eukaryotic −1PRF signal would also be fascinating. While the central features and mechanisms of 70S and 80S ribosomes are conserved, almost without exception, the present 70S studies utilize slippery heptamers ending in AAG, decoded by a modified lysine tRNA, which is frameshift prone and promotes an unusual single-tRNA slippage event, whereas asparaginyl-tRNA (decoding AAC) promotes dual-tRNA slippage (150, 151). Studies should also consider the effects of other *cis*- and *trans-*acting factors beyond the minimal element. A general concern in the field is that many −1PRF assay systems are heterologous, often flanking the signal with unrelated reporter genes. This can obscure important contributions from distal elements and lead to unexpected, reporter-derived artifacts. The importance of context in −1PRF has been discussed recently in an excellent review (152).

Further work is also needed to explain unexpected findings. Desai and colleagues (120) have shown that mRNA hairpin opening and translocation occur concurrently, and not simultaneously with EF-G arrival via the free energy gained upon binding. This indicates that GTP hydrolysis provides the energy for unwinding, questioning the role of the mRNA channel helicase, thought to be the first region of contact with the stimulatory RNA. Conceivably, contact of the stimulator elsewhere with the ribosome might prime the ribosome for unwinding at the entry channel when the power stroke is delivered. By decreasing the force applied to the ends of a hairpin-containing mRNA to simulate a more stable barrier to elongation, these authors have also observed that ribosomes switch to a sixfold slower alternative kinetic pathway, or slower gear (120; as also described more recently in 153), perhaps to better exploit thermal fluctuations in RNA structure, minimizing energy dissipation. The relevance of these observations to −1PRF needs further investigation.

Why and when viruses utilize frameshifting might appear self-evident; in most cases so far, it is an efficient way to produce defined ratios of products throughout infection. However, there remains more to discover. There is already evidence that RNA- and protein-stimulated −1PRF signals can be tuned to be more or less active at different points in the replication cycle, and we do not know the extent to which other viral and cellular factors might modulate frameshifting. The significant effect of amino acid substitutions in the nascent peptide on −1PRF efficiency at the SINV signal supports a potential link between frameshifting and virus evolution (106). Indeed, the role of −1PRF in virus replication cycles is understudied at present. Given the huge RNA virome, it is unquestionable that new −1PRF signals will be discovered in viruses, and with the understanding that stimulatory elements can go beyond the core signal, it is likely that more examples in both viral and cellular genes will be identified and new mechanisms unearthed (154). There is growing evidence that ribosome density on an mRNA can influence frameshifting, not only through RNA refolding kinetics but also via ribosome collisions (155). Further, Lyon and colleagues (156) propose that frameshifting might vary on an mRNA population level, with some individual mRNAs predisposed to frameshift at very high efficiencies, and others hardly at all.

The study of –1PRF will continue to excite and challenge researchers. The field has come a long way since the early studies of Jacks & Varmus (6), yet there is still much to be discovered, in both viruses and their hosts.
